# Epidermoid Cyst of the Tongue—A Rare Case

**DOI:** 10.5005/jp-journals-10005-1594

**Published:** 2019

**Authors:** Mayuri Jain

**Affiliations:** Department of Oral Medicine and Radiology, Maharana Pratap College of Dentistry and Research Centre, Gwalior, Madhya Pradesh, India

**Keywords:** Epidermoid cyst, Oral cavity, Pathogenesis, Tongue, Ultrasonography

## Abstract

**Introduction:**

Epidermoid cysts are commonly found in various part of the body, but its occurrence in the oral cavity is relatively rare.

**Case presentation:**

Present case highlights the importance of involvement of epidermoid cyst within the tongue in 10-year-old child.

**Discussion:**

The occurrence of epidermoid cyst within the tongue is an extremely rare phenomenon and only few cases have been reported in the literature. Clinically they are painless, slowly growing mass causing difficulty in breathing, eating and swallowing Imaging modalities most importantly; ultrasonography helps in the evaluation of the lesion. However, histopathology remains the mainstay for definitive diagnosis in such conditions.

**Conclusion:**

From the present case, it is concluded that such type of cases needs to be reported as it highlights the importance of keeping the rarities in mind along with common lesions while making a diagnosis.

**How to cite this article:**

Jain M. Epidermoid Cyst of the Tongue—A Rare Case. Int J Clin Pediatr Dent 2019;12(1):80–82.

## CASE DESCRIPTION

**Figs 1A and B:**
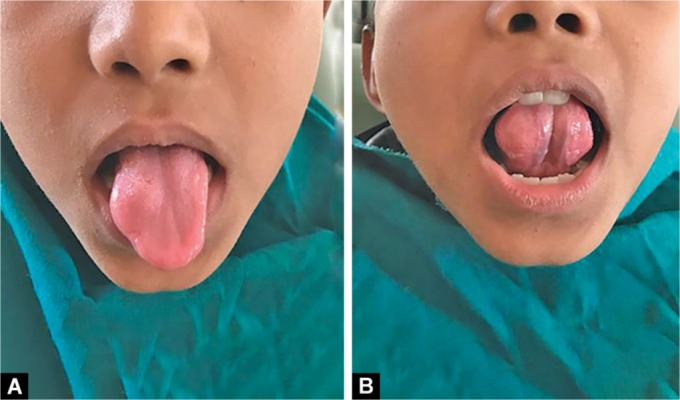
Intraoral photograph showing round swelling in right side of dorsum surface of tongue (A) extending up to the ventral surface (B)

**Fig. 2:**
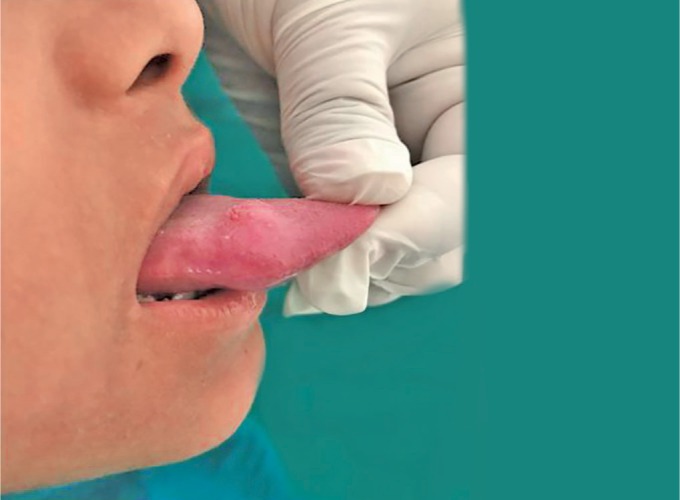
Intraoral photograph showing round swelling measuring 2.5 × 2.5 cm in size extending up to lateral surface of tongue

**Fig. 3:**
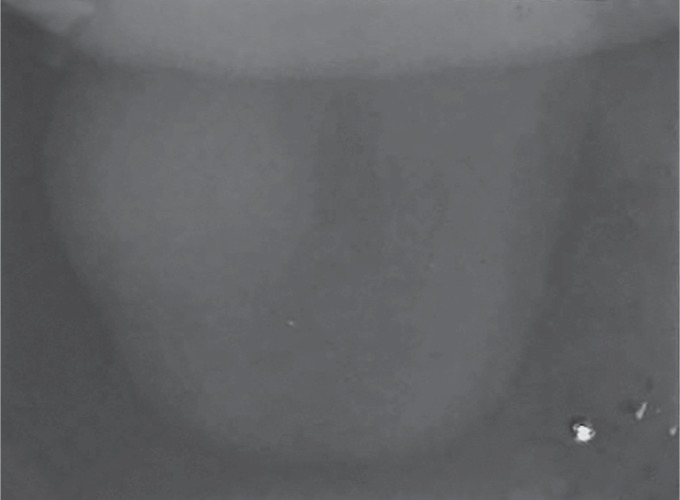
Intraoral periapical radiograph showing the lesion did not reveal any calcifications

**Fig. 4:**
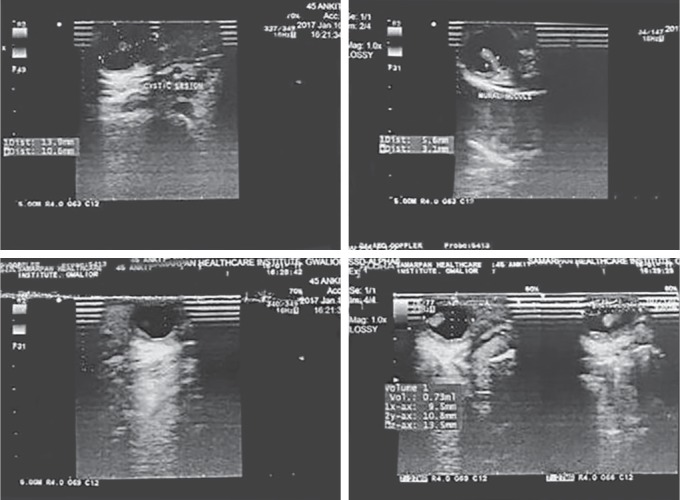
Ultrasonography of the lesion revealed a thin walled anechoic cystic lesion measuring 13 × 10 × 9 mm in the submucosal plane

**Fig. 5:**
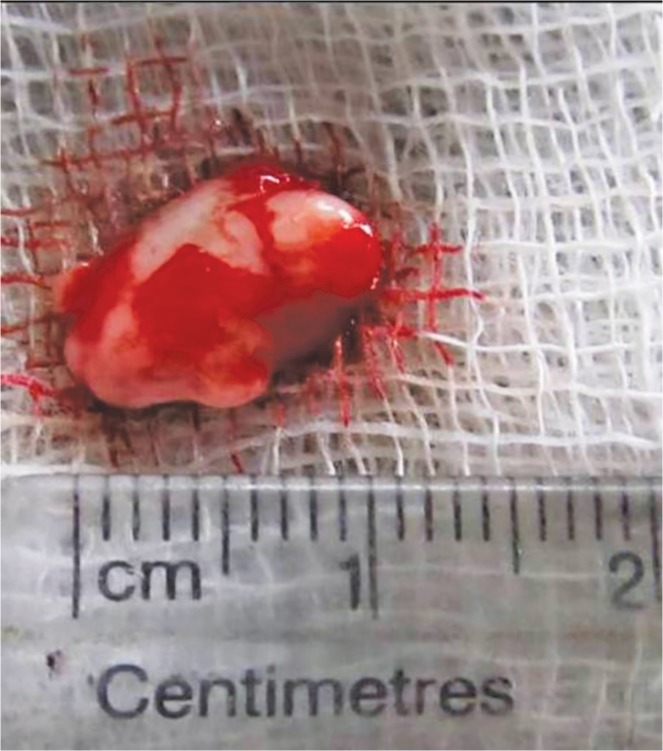
Excised specimen of size 3 × 3 cm

**Fig. 6:**
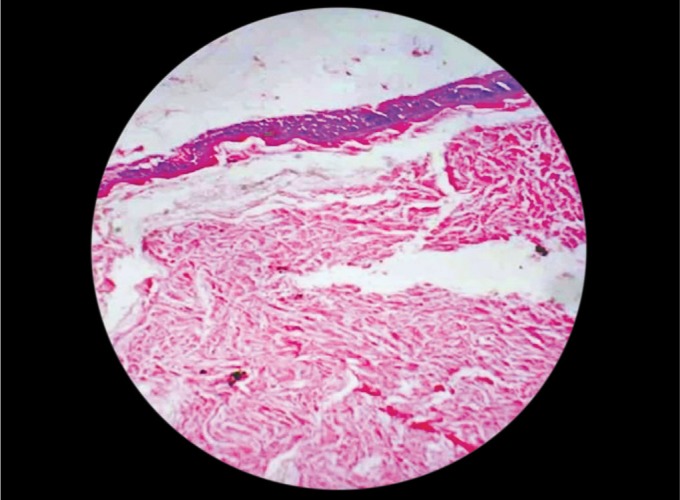
Photomicrograph of the lesion showing keratinizing squamous epithelium with distinct granular layer (H and E stain; magnification ×40)

A 10-year-old male child patient reported at the Department of Oral Medicine and Radiology with a chief complaint of swelling on the anterior part of the tongue since 3–4 months. The swelling was small in size and progressed gradually over the time to reach the present size. The patient has no history of pain; however, discomfort was present. This was associated with difficulty in speech and mastication as the swelling increased in size of the cyst. The past medical and dental history was not significant. General physical examination and extraoral examination were unremarkable. Intraoral examination revealed a well-circumscribed, round, firm, non-tender, cystic swelling of about 2.5 × 2.5 cm in size, with a smooth surface (Fig. 1). Overlying mucosa looked normal. Swelling was embedded in the right side of the dorsum surface of the anterior tongue involving the lateral border and extending up to the ventral surface (Figs 1 and 2). There was no discharge from the swelling. The swelling was asymptomatic. Based on the history and clinical appearance of the lesion, a provisional diagnosis of the dermoid cyst was hypothesized. Differential diagnosis of the lesion included epidermoid cyst and lymphoepithelial cyst. The investigations included complete hemogram and intraoral periapical radiograph and ultrasonography. Routine hematological investigation values were found to be within normal limits. No calcification was detected on an intraoral periapical radiograph (Fig. 3). The ultrasonic scan of the lesion revealed a thin-walled anechoic cystic lesion measuring 13 × 10 × 9 mm in the submucosal plane of anterior one-third of the tongue on the right superior aspect. The lesion did not show internal vascularity; however, there was a mural nodule measuring 5.6 × 3.1 mm which demonstrates faint vascularity (Fig. 4). Complete surgical excision of the cyst was done and the mass was sent for histopathological examination (Fig. 5). Histopathology confirmed the diagnosis of an epidermoid cyst by the presence of the stratified squamous epithelial tissue covering the cyst cavity, with laminas of parakeratin on the surface (Fig. 6). The postsurgical period was completely successful. The patient was followed-up for the next 6 months, and no recurrence of the lesion was observed.

## DISCUSSION

Epidermoid cyst is very rare nonodontogenic cystic lesions with unknown etiology.^1^ It is a congenital cyst which is derived from the germinal epithelium. This cyst may also be acquired or secondary due to the insertion of epidermal elements into the dermis posttraumatically or iatrogenically, so it has also been termed as the epidermal inclusion cyst.^2^ Epidermoid cyst has to be differentiated from the dermoid cyst by the lack of skin adnexa such as hair follicles or sebaceous gland, which is present in the dermoid cyst. These lesions are more frequent in young adults and rarely occur in infants,^3^ but, in our case, it was present in a child. These cysts are generally solitary lesions.^4^ Epidermoid cyst has been found throughout the body, out of them, only 1.6% has been found in the oral cavity.^5^ Out of all oral cystic lesions, the epidermoid cyst represents less than 0.01%,^6^ but, in the present case, the cyst was seen in the oral cavity. In the oral cavity, they are commonly seen on the floor of the mouth,^7^ but, in the present case, the cyst was seen within the tongue. Epidermoid cyst present within the tongue is very rare entities. In the tongue region, these lesions may be formed by remnants of the tuberculum impar.^8^ A review of literature indicates that various cases of epidermoid/dermoid cyst affecting extraorally have been reported but only few cases have been reported affecting the oral cavity. In the oral cavity, only a few cases affecting the floor of the mouth have been reported. Shore reported only four cases of sublingual dermoid cyst in a review of 54,000 cases.^9^ Taylor et al. in their study found that a total of 541 evident dermoid cysts of the body, in which, 184 (34%) were present in the head and the neck and 35 (6.5%) of these on the floor of the mouth.^10^ New and Erich in a review of 1,459 cases of the dermoid cyst at Mayoclinic found that only 16% of cases involved the oral cavity and, among them, only one-fourth arose from the floor of the mouth.^11^ Another study of Taylor et al. found that only 65% of cases of the dermoid cyst were intraoral.^12^ Till date, only one or two cases of epidermoid cyst affecting the tongue have been reported. Here, we report a rare case of epidermoid cyst affecting the tongue. The clinical feature of the lesion is not characteristic and only consists of cystic swelling. As their size enlarges, functional problems such as speech articulation, mastication, deglutition, and respiration can be expected to occur.^1,8^ Ultrasonography at best can be considered as an outstanding imaging modality for accurate localization, detection, and extent of the lesion with surrounding structures. However, histopathology remains the mainstay for definitive diagnosis in such conditions. The treatment includes the complete surgical excision.^1,8^ Recurrences are very rare. This case is unique because after histopathology, diagnosis of the epidermoid cyst has been arrived at, which was one of the rarest diagnoses at this location.

## CONCLUSION

Epidermoid cysts in the tongue are quite rare and need to be differentially diagnosed from numerous other diseases and conditions of the area. The definitive confirmation and diagnosis of the disease is always affected *via* histopathology. From the present case, it is concluded that such type of cases needs to be reported as it highlights the importance of keeping the rarities in mind along with common lesions while making a diagnosis.
